# Thermography in the diagnosis of carpal tunnel syndrome

**DOI:** 10.1515/med-2021-0007

**Published:** 2021-01-27

**Authors:** Piotr Bargiel, Norbert Czapla, Piotr Prowans, Daniel Kotrych, Paweł Ziętek, Dariusz Lusina, Paweł Łęgosz, Jan Petriczko

**Affiliations:** Department of Plastic, Endocrine and General Surgery, Pomeranian Medical University, ul. Unii Lubelskiej 1, 71-252, Szczecin, Poland; Department of Orthopaedics, Traumatology and Orthopaedic Oncology, Pomeranian Medical University, Szczecin, Poland; Department of Orthopaedics and Traumatology, Medical University of Warsaw, Warsaw, Poland

**Keywords:** dynamic thermography, sympathetic system, carpal tunnel

## Abstract

**Introduction:**

Carpal tunnel syndrome (CTS) is a condition caused by chronic compression of the median nerve. The diagnosis is made mainly on the basis of clinical image and confirmed with electrodiagnostic testing (electromyography and nerve conduction study); however, these methods do not always aid in reaching the diagnosis of CTS. Moreover, they are invasive examinations, unpleasant for the patient and have to be performed by a qualified physician.

**Aim:**

An evaluation of the usefulness of dynamic thermography in the diagnosis of CTS.

**Material and methods:**

Forty patients were included in the study group. CTS was diagnosed based on clinical examination and electromyography. Forty healthy volunteers were included in the control group. Each of the participants was examined thrice with dynamic thermography. The patient’s hands were first cooled down and then a thermal camera measured their return to normal temperature. The measurement was repeated on the dorsal and volar aspects of each hand.

**Results:**

The results obtained in the study show that a relief of symptoms after carpal tunnel release does not correlate with thermal image. Moreover, the return to normal hand temperature was faster in the control group. In patients with unilateral CTS, no difference was observed in thermographic images of the affected and healthy hands.

**Conclusions:**

1. Dynamic thermography can be useful in confirming CTS diagnosis.
2. Dynamic thermography does not allow for objective assessment of patient’s complaints in the postoperative period.
3. This method has currently limited clinical application. Due to complexity, it presently serves mainly scientific purposes.

## 1 Introduction

Carpal tunnel syndrome (CTS) is caused by chronic compression of the median nerve within the carpal tunnel. It is one of the most common peripheral mononeuropathies and the most common upper extremity neuropathy. Prevalence in general population is estimated at 0.1–1%. Risk factors include female gender and physical labour. Ten percent of patients have bilateral CTS [1,2,3].

The diagnosis is based on clinical image. Patients complain about nocturnal pain and paresthesia, which wake them up during the night and as the disease progresses, the symptoms increase in severity and begin to appear during the day. In advanced cases of CTS, motor disorders occur in the form of grip precision and strength loss. It makes handling small items (such as a pen or keys) more difficult. Thenar muscle atrophy is observed on examination. Due to decreased or abundant sensitivity, some patients develop wounds on fingertips from repetitive trauma [4,5,6,7].

Clinical presentation is not always obvious. In some cases, one group of symptoms can dominate for years. There are patients with considerable motor deficits without pain or sensitivity impairment and patients with positive electrophysiological results who, despite being in severe pain for years, have no muscle atrophy. This varying clinical image could be a result of overlap syndromes, multifocal compression on the median nerve (“double crush” syndrome) or substitution of lost median nerve functions by the ulnar nerve [8].

Electrodiagnostic testing plays a key role in diagnosing peripheral nerve disorders. Nerve conduction study (NCS) and electromyography (EMG) are mostly used when CTS is suspected. NCS directly measures the sensory and motor fibre conduction speed and amplitude, while with EMG it is possible to register and assess the function of thenar muscles (it has a lower sensitivity than NCS). Many clinicians consider these electrodiagnostic examinations as gold standard to diagnose CTS; however, it has been proved that approximately 18% of patients with clinical signs of median nerve compression have unimpaired nerve conduction in a classic NCS examination. Both NCS and EMG are invasive, unpleasant for the patient and a qualified physician must perform them. Moreover, according to many authors, they are not more sensitive than physical examination combined with medical history [8,9,10,11].

In search of new objective CTS diagnostic tests, researchers have recently taken interest in thermography.

Infrared thermography (IRT, IR) records a map of surface temperature of an examined area. Skin temperature indirectly reflects blood supply to a given area, which changes in response to various stimuli. Such measurements are easy, non-invasive, inexpensive and quick. These features make IRT a promising diagnostic test [12,13,14] (Tables 1 and 2).

**Table 1:** Skin temperature change in the study group (volar and dorsal side) on days −1, 1 and 14 after 3 min cold exposure expressed as area under the curve

Study group												
No	Dorsal aspect (d)						Volar aspect (V)					
	Right (R)			Left (L)			Right (R)			Left (L)		
	Day −1 [cm^2^]	Day 1 [cm^2^]	Day 14 [cm^2^]	Day −1 [cm^2^]	Day 1 [cm^2^]	Day 14 [cm^2^]	Day −1 [cm^2^]	Day 1 [cm^2^]	Day 14 [cm^2^]	Day −1 [cm^2^]	Day 1 [cm^2^]	Day 14 [cm^2^]
1	5738.7954	7661.8318	5247.0674	5830.4074	7809.6044	5044.512	5733.7871	7407.9383	5139.8895	5588.9585	7955.766	4949.2093
2	6788.1167	6406.5961	5278.8475	6001.192	6048.0365	5677.8629	6696.8626	6618.3152	6134.609	6148.8008	7393.307	6040.4161
3	7047.3592	6509.9462	6942.9268	6964.1448	6306.3424	6891.9942	7077.0337	6199.914	6981.5185	7094.2211	6912.317	6540.6793
4	7172.5313	7559.0131	7628.7504	7416.5034	7743.7833	7604.5627	7005.7044	7346.557	7493.6902	7381.5325	7581.901	7526.9028
5	7407.8042	7601.5353	7517.0499	7430.2092	7605.3407	7595.3287	7709.701	7397.9859	7515.4516	7735.8987	7873.257	7565.0939
6	6817.0816	7593.8763	7474.5757	6558.6345	7540.5374	7370.4258	6687.5851	7295.9813	6926.9493	6494.2285	7620.692	7109.7913
7	8015.8948	6642.3724	7418.5549	8067.7989	7265.3256	7310.884	7934.782	6723.329	7081.0221	7946.9858	7759.517	7324.1033
8	7309.7887	7367.0289	4256.9423	6955.7899	7163.2455	4254.8952	6534.5163	7515.998	4194.0041	6346.3395	7142.091	4337.0405
9	7582.8238	6414.475	6377.498	7258.309	6216.576	5503.479	6910.09	6295.712	5788.669	6437.497	7375.88	5323.079
10	7466.418	7371.716	6050.844	7289.279	6507.579	5578.934	6941.636	7108.365	5826.119	6324.852	7215.417	5412.285
11	5566.186	5214.194	7282.395	5348.346	4986.756	7250.772	5536.749	5125.916	7612.315	5254.351	6564.692	7540.743
12	7333.067	7260.516	7506.669	7447.416	7341.889	7426.07	7426.768	7736.378	7506.669	7626.45	6309.18	7552.422
13	5784.216	6162.626	7575.474	6053.251	5746.267	7581.58	6743.047	6050.695	7499.184	6991.285	5785.055	7293.785
14	7711.548	6727.103	7468.428	7621.656	6704.846	7495.8	7666.691	6510.624	7646.282	7675.845	7615.244	7687.339
15	6903.785	7140.782	7581.512	6908.295	7040.08	7675.327	7107.863	7051.307	7629.842	7255.583	6771.736	7769.634
16	7760.978	0	0	7660.777	0	0	7579.234	7522.356	0	7632.886	7931.145	0
17	7720.041	7547.857	7649.1	7807.966	7597.292	7653.915	7435.501	7735.052	7708.1	7581.559	7378.262	7799.344
18	6552.423	5099.829	0	6583.997	4957.659	0	5284.565	5811.949	0	5579.183	6486.184	0
19	7378.214	7450.177	6580.865	7493.246	7627.63	6927.553	6337.277	7625.306	6572.66	6579.345	7543.51	6909.17
20	6925.756	7307.599	7673.456	6658.792	6635.398	7319.403	7591.466	6981.113	7636.585	6018.125	7854.768	7572.801
21	7377.338	7467.43	7931.33	7212.892	7619.24	7897.262	6986.53	6553.641	7629.767	7159.964	7601.74	7608.069
22	6823.587	7226.326	7692.785	6554.609	7292.132	7522.476	7097.08	7210.87	7736.019	7088.723	7770.488	7575.838
23	7188.357	5547.25	8012.986	7049.089	5376.11	7894.202	7118.68	5604.277	7858.293	6936.609	7138.907	7633.78
24	7384.856	7384.335	8043.201	7368.208	7416.252	8101.438	7368.408	7385.482	8052.101	7433.914	7325.788	8068.624
25	7641.064	7615.568	8147.866	7619.311	8019.349	8167.685	7561.372	7561.845	8023.069	7609.731	7192.348	8051.5709
26	7363.349	0	7491.52	7365.141	0	7581.078	7217.851	0	7649.151	7358.705	6068.448	7719.987
27	7465.357	7229.305	7940.297	7385.472	7322.485	7896.825	7453.162	7255.292	7536.237	7450.363	6928.541	7611.418
28	7453.065	7723.253	7597.0091	7583.082	7777.943	7660.194	6325.129	7698.297	7341.56	6549.858	7325.788	7342.615
29	6189.559	7204.995	7194.602	6348.549	7094.84	7054.25	6312.377	7321.995	6186.51	6041.102	5812.128	5834.426
30	7292.017	7177.755	7637.219	7352.46	7318.195	7731.399	6794.774	7168.356	7498.795	6947.294	7592.575	7562.134
31	7536.452	0	6345.166	6345.166	0	6102.38	7163.624	0	6445.802	7217.876	6748.843	6327.314
32	7443.36	7578.313	7383.942	7383.942	7691.162	7571.457	7481.491	7562.951	7472.296	7558.933	7940.122	7614.151
33	7584.729	6402.215	6326.16	6326.16	6266.061	5480.812	6925.2831	6424.963	5771.308	6470.631	7479.213	5372.737
34	7245.426	7352.746	4117.947	4117.947	7099.679	4322.886	6658.887	7597.089	4210.107	6492.073	7073.213	4488.695
35	6734.632	5518.511	6959.144	6959.144	5773.442	6953.582	6343.529	5743.148	7357.987	6001.186	7445.154	7240.555
36	6926.741	7615.272	7182.663	7182.663	7598.465	7139.323	6628.539	7448.668	7266.991	7091.038	7361.571	7425.18
37	6928.644	7549.101	7618.502	7618.502	7602.188	7586.2975	7028.173	7303.756	7498.523	7421.219	6441.04	7516.028
38	6607.199	6165.755	6805.185	6805.185	6474.394	6896.919	5455.211	6185.663	6499.905	6386.031	7575.476	6100.129
39	5617.811	5752.141	6222.673	6222.673	6836.857	6986.948	5754.189	5461.753	5198.507	5922.529	7862.005	6047.83
40	5505.797	5750.37	5240.434	5240.434	6015.909	6184.999	5572.637	5892.825	5199.465	5368.412	7594.724	6065.676

**Table 2:** Skin temperature change in the control group (volar and dorsal side) on days −1, 1 and 14 after 3 min cold exposure expressed as area under the curve

Control group												
No	Dorsal aspect (d)						Volar aspect (V)					
	Right (R)			Left (L)			Right (R)			Left (L)		
	Day −1 [cm^2^]	Day 1 [cm^2^]	Day 14 [cm^2^]	Day −1 [cm^2^]	Day 1 [cm^2^]	Day 14 [cm^2^]	Day −1 [cm^2^]	Day 1 [cm^2^]	Day 14 [cm^2^]	Day −1 [cm^2^]	Day 1 [cm^2^]	Day 14 [cm^2^]
1	7931.789	8018.725	7992.002	7944.69	8021.499	8001.998	7837.452	7958.452	7902.098	7818.496	7955.766	7880.987
2	7420.906	7807.555	7549.036	7157.381	7746.42	7730.55	6942.505	7507.791	7332.101	6824.457	7393.307	7540.394
3	7375.576	7242.062	7417.303	7275.891	7331.754	7442.08	7019.673	6717.26	7004.259	6846.933	6912.317	7107.129
4	7496.062	7470.305	7660.607	7506.423	7573.31	7646.501	7499.088	7363.305	7381.77	7420.631	7581.901	7322.57
5	7838.856	7801.766	7576.021	7928.603	7777.979	7605.85	7762.567	7867.767	7272.483	7799.8	7873.257	7269.62
6	7841.396	7797.629	7802.432	7831.478	7830.348	7830.986	7895.997	7592.659	7792.098	7955.076	7620.692	7823.987
7	7939.365	7956.175	8003.475	7902.297	7933.434	7974.1	8078.802	7840.773	7901.058	7968.235	7759.517	7858.535
8	7785.669	7395.189	5898.385	7814.308	7497.196	5924.191	7509.795	7032.656	5761.783	7491.527	7142.091	5779.267
9	7847.208	7481.591	6979.941	7851.236	7499.11	7029.309	7729.167	7280.531	6881.055	7716.017	7375.88	7043.586
10	8122.939	7690.557	7379.837	8006.076	7628.331	7241.683	7707.437	7308.749	7069.012	7691.005	7215.417	7027.306
11	5484.605	6107.246	5430.307	5578.629	6284.529	6148.846	5498.959	6392.72	6350.993	5658.332	6564.692	6441.04
12	7614.6	6932.045	6589.296	7349.413	6972.879	6887.417	7304.864	6614.023	6122.468	6625.497	6309.19	6307.162
13	5475.814	5996.919	5927.656	5434.092	6087.442	5353.154	5020.157	5820.822	5845.526	4909.604	5785.055	4855.228
14	7521.972	7581.084	7577.265	7556.966	7598.959	7548.5	7507.015	7598.077	7578.142	7461.083	7615.244	7592.575
15	7256.8412	7289.844	0	7198.52	7216.869	6171.461	6818.311	6876.332	6605.62	6775.314	6771.736	6455.032
16	7938.222	8006.154	0	7936.055	8003.07	0	7784.173	7977.427	0	7806.163	7931.145	0
17	7347.619	7810.582	7524.392	7047.287	7680.187	7691.494	6962.052	7474.044	7382.423	6835.164	7378.262	7476.944
18	5426.226	6037.146	5431.891	5520.76	6188.335	6262.346	5367.018	6289.378	6502.053	5541.145	6486.184	6518.376
19	7484.808	7475.43	7647.502	7493.637	7551.137	7526.288	7444.353	7392.796	7385.374	7386.396	7543.51	7359.638
20	7809.787	7750.991	7578.268	7884.112	7692.559	7567.222	7769.421	7822.063	7251.109	7747.183	7854.768	7287.472
21	7838.235	7819.318	7817.969	7838.25	7809.712	7774.923	7880.007	7587.374	7640.158	7659.061	7601.74	7714.518
22	7933.339	7914.865	8006.161	7916.831	7931.969	7957.458	8106.917	7829.728	7907.318	7986.147	7770.488	7871.523
23	7791.83	7408.545	8536.373	7808.404	7457.339	5828.149	7520.686	7102.784	5671.876	7466.99	7138.907	5686.76
24	7836.506	7447.504	6963.893	7791.804	7434.288	6945.826	7718.714	7251.486	6889.445	7733.469	7325.788	6995.476
25	8127.782	7681.88	7438.169	8095.436	7627.691	7347.806	7709.021	7287.42	7024.735	7681.524	7192.348	6954.057
26	7424.791	6589.296	6703.144	7033.306	6652.372	6710.664	6982.44	6296.018	6505.96	6307.162	6068.448	6200.716
27	7334	7250.992	7461.055	7421.676	7362.357	7411.68	7051.114	6795.498	7034.848	6856.861	6928.541	7088.462
28	7836.506	7447.504	6963.893	7791.804	7434.288	6945.826	7718.714	7251.486	6889.445	7733.469	7325.788	6995.476
29	5452.463	5983.178	5907.96	5353.154	6054.229	5218.644	4904.129	5845.526	5649.476	4869.233	5812.128	5166.471
30	7542.093	4577.265	7552.235	7548.5	7567.339	7516.741	7557.708	.837	7560.877	7495.211	7592.575	7528.427
31	7290.9382	7252.433	6430.989	7153.462	7154.4	6097.115	6801.685	6807.516	6574.856	6748.019	6748.843	6446.622
32	7983.932	7998.098	7930.323	7789.432	8009.213	8013.3344	7839.432	7900.321	7889.423	7870.321	7940.122	7789.212
33	7400.433	7840.211	7398.321	7148.321	7534.432	7723.212	6930.213	7309.213	7430.21	6789.432	7479.213	7254.423
34	7349.234	7238.245	7381.23	7361.321	7382.234	6990.321	7092.543	6982.342	7150.543	6892.12	7073.213	7321.34
35	7564.423	7423.44	7790.21	7440.221	7543.133	7612.309	7545.234	7289.453	7432.432	7632.12	7445.154	7333.905
36	7203.645	7749.725	7626.76	7020.433	7614.456	7658.371	7014.062	7503.886	7374.112	6751.403	7361.571	7420.704
37	5430.307	6068.343	5415.025	5523.639	6148.846	6294.398	5415.601	6350.993	6472.183	5541.175	6441.04	6488.674
38	7516.488	7530.994	7674.995	7507.107	7589.853	7513.501	7444.353	7403.291	7425.145	7422.26	7572.476	7399.222
39	7804.203	7720.468	7577.005	7858.586	7680.731	7568.589	7803.775	7812.666	7276.477	7776.551	7862.005	7327.217
40	7817.696	7819.385	7817.696	7850.02	7776.581	7681.169	7894.325	7575.491	7727.383	7966.299	7594.724	7827.202

Few studies evaluating thermography in CTS have been published. Furthermore, no measurement standard is presently available [15].

## 2 Aim

An evaluation of the usefulness of dynamic thermography in the diagnosis of CTS.

## 3 Material and methods

### 3.1 Material

Out of 84 patients who underwent carpal tunnel release in our department in years 2014–2016, 40 were included in the study group – 32 females and 8 males aged 32 to 68 (mean age 54 years). Diagnosis was based on clinical examination and EMG. Symptoms had to present for a minimum of one year, mean duration was 21 months. Ten patients had bilateral CTS. Right hand was affected in 29 cases, left hand in 11 patients. Thirty-two patients were right-handed, 8 patients left-handed.

Control group consisted of 40 healthy volunteers: 25 women and 15 men aged 27 to 63, mean age was 49 years. The right hand was dominant in 34 people, the left hand in 6. No member of the control group complained on any hand, cervical or thoracic spine disorders.

### 3.2 Methods

Study protocol was approved by the Pomeranian Medical University Ethics Committee (KB-0012/94/13). Patients included in the study had no significant comorbidities. Smokers and patients with carpal release or carpal tunnel injections were excluded from the study. Each patient was examined with dynamic thermography thrice. First examination was performed one day prior to the surgery, second a day after surgery and the third one 2 weeks after surgery.

All measurements were taken in a room with a steady temperature of 22°C and 65% humidity. Patient’s hands were placed on a table 20 cm away from the camera and fan, which acted as a cooling device. Hands were cooled down for 20 s. A FLIR T335 camera was used to obtain thermal images after the fan was stopped. Measurements were sampled every 5 s for 3 min. The dorsal aspect was assessed first, then after re-cooling, the volar side. To analyse the thermographic images, FLIR Research IR programme was used. Temperature values were sampled from the fingertips (on the volar side) and nails (on the dorsal side) of fingers I-III. Capillary circulation efficiency was measured as area under curve in a graph that showed temperature change in time.

## 4 Results

Patients from the study group had undergone carpal tunnel release. At 24 h postoperatively, 37 patients reported spontaneous pain mitigation (which previously woke them up), while 3 patients did not report any improvement. At 14 days postoperatively, symptoms subsided in 39 patients, while one reported an exacerbation of pain.

**Figure 1:**
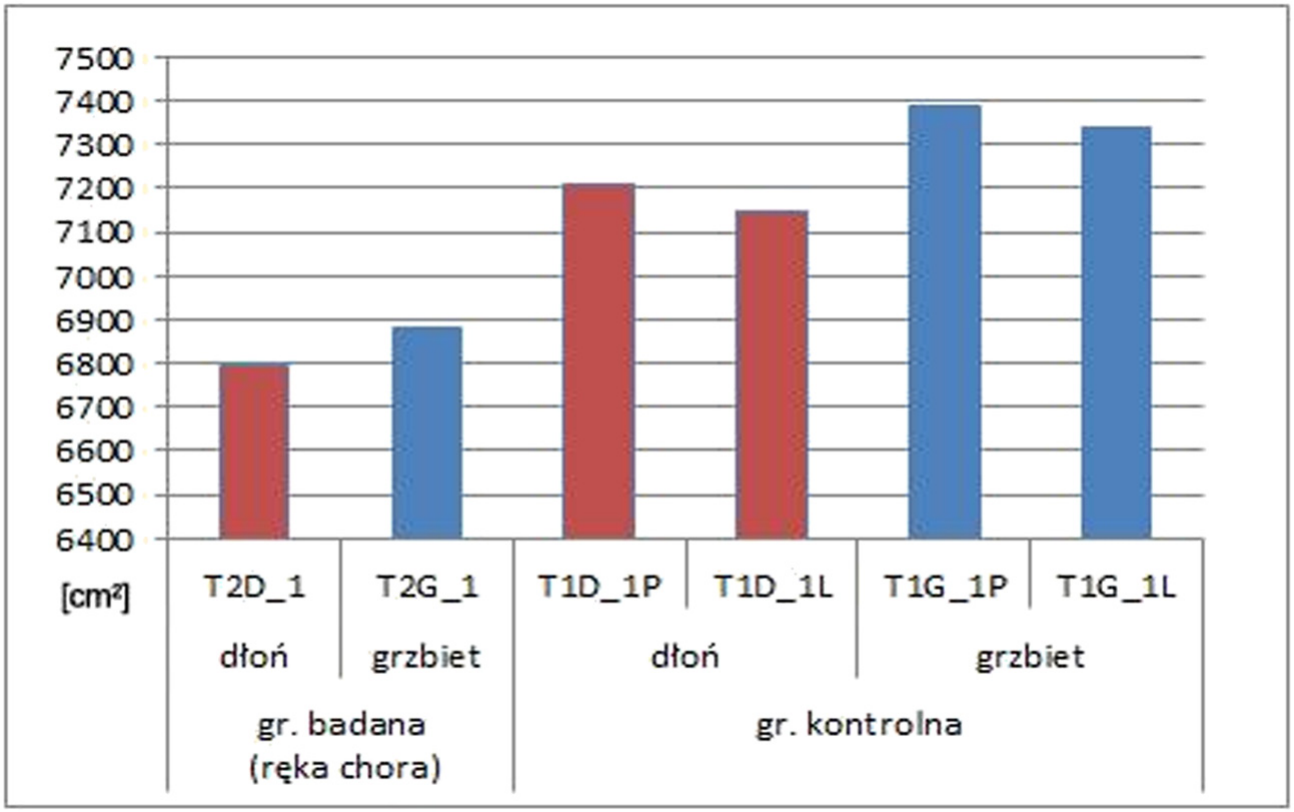
A comparison of temperature change between the affected (operated) hand in the study group and both hands of control group.

During dynamic thermography, it was observed that after the cold stimulus had been withdrawn, temperature rose during the first 2 min and then stopped. Therefore, only data gathered during these first 2 min were included in the statistical analysis (Figure 1).

## 5 Discussion

In patients with median nerve compression, it appears natural that apart from motor and sensory fibre damage, autonomic nerve fibres can also be affected. Clinical examination and electrophysiological testing both measure the extent of motor and sensory function loss. Some patients, however, complain about symptoms resembling Raynaud’s phenomenon. Trophic changes to the skin are rare, although possible [16,17]. Literature data show that even as many as 50% of patients with CTS may suffer from sympathetic dysfunction [18].

In the study group, 75% of patients reported symptoms related to the sympathetic system: sweating, dryness, pallor, rubor, swelling or cyanosis. Literature reveals many papers where the extent of sympathetic system damage in CTS is evaluated by plethysmography, capillaroscopy or Doppler ultrasound. Another method is sympathetic skin response (SSR), where the electric potential of the skin varies depending on surface temperature, which reflects the activity of sweat glands controlled by the sympathetic nervous system [19,20,21,22,23,24].

While analysing the results, a number of questions had been posed.

First, does the thermographic image of a healthy hand (control group) differ from the image of an affected hand prior to the surgery?

There was a statistically significant difference in thermographic images of hands of patients from both groups. In CTS, blood flow is higher, because the return to original temperature occurs quicker. Based on this observation, it can be assumed that dynamic thermography can be used to determine if a patient is suffering from CTS. Similar conclusions were made by other authors. Papez and Palfy compared the results of static thermography with NCS in patients with CTS. Control group was examined first to create a thermographic model of a healthy hand and then, using an artificial neural network, this model was compared with a thermographic image of patients affected by CTS. The results of that study were statistically significant – authors concluded that thermography is a tool with high sensitivity and is useful in diagnosing severe cases of CTS. When patients with less exacerbated CTS were included into the study group, the sensitivity, however, was lowered. In our study, most patients were diagnosed with moderate CTS (stage II, according to NCS). Papez also observed during his study that the sensitivity of the examination was higher on the dorsal side of the hand. [25,26,27]. Similar conclusions were made by Zivcak et al. [28]. Some authors examined only the volar aspect of the hand [29,30,31]. In our study, both the volar and dorsal sides of the hand were evaluated, and the results show a significant difference in blood flow for both sides between the study and the control group.

Secondly, do thermographic images of the unaffected hand in CTS patients in three examinations (a day before the surgery, one day after the surgery and 14 days after the surgery) differ among each other? In unilateral CTS, thermographic images of the unaffected hands did not differ in consecutive examinations. Similarly, 3 examinations of control group volunteers did not show any differences. Such stable thermographic images confirm the reproducibility of the method.

Thirdly, does the relief of symptoms during the first day after surgical carpal tunnel release correlate with changes in thermographic images?

Immediate relief of nocturnal pain after carpal tunnel release is seen in most patients with CTS. In this study, a similar postoperative observation was made in nearly all patients. It did not, however, correlate with thermographic images captured in 24 hours and 14 days after the surgery. In literature, there is scarce information on changes in blood circulation in patients with CTS after surgical release. A similar study had been conducted by Z. Ming and J. Sivola in 2007. Static thermography had been used to evaluate capillary flow. The diagnosis of CTS was made purely on NCS, without taking the clinical image into consideration. The first thermographic image was captured prior to the surgery, the second 6 months after. In our study, carpal tunnel was diagnosed mainly on signs and symptoms with NCS results only to confirm the diagnosis. A lack of NCS result with a clear clinical image of CTS did not exclude a patient from the study. Ming et al. in their study showed that an improvement in circulation follows a carpal tunnel release within 6 months. Immediate relief of nocturnal symptoms is related to an increase of blood flow to the nerve itself, while full healing is achieved after 6 months due to reinnervation [29].

Lastly, does a thermal image of the affected and unaffected hand differ in the same patient with CTS?

A comparison of the affected and unaffected hand in the same patient with CTS did not reveal statistically significant differences in hand temperature. This may be a result of many overlapping factors. CTS is diagnosed with clinical image. NCS is an additional examination to confirm the already suspected diagnosis and to evaluate the extent of nerve damage. Healthy limbs in patients with CTS were described as those showing no clinical symptoms, not ones with a negative NCS result. Out of 40 study group patients, as few as 12 people had a bilateral NCS, and in that group, only 2 patients had CTS excluded. Literature data estimate the prevalence of bilateral CTS at 50%. Not all symptoms of early CTS could be noticed by a patient. Thus, it may be possible that among 23 healthy limbs, some may be in an early stage of the disease. Most researchers compared hands with confirmed CTS with healthy limbs from the control group. Few authors focused on looking upon both hands of the same patient. The majority assessed the temperature difference of two corresponding points on the volar and dorsal aspect and then compared the results to the control group [25,26,27,28].

## 6 Results

Our research shows that:

1. Dynamic thermography can be useful in confirming CTS diagnosis.
2. Dynamic thermography does not allow for objective assessment of patient’s complaints in the postoperative period.
3. This method has currently limited clinical application. Due to complexity, it presently serves mainly scientific purposes.

## References

[1] Mondelli M, Giannini F, Giacchi M. Carpal tunnel syndrome incidence in a general population. Neurology. 2002;58:289–94. 10.1212/wnl.58.2.289. 11805259

[2] Ibrahim I, Khan WS, Goddard N, Smitham P. Carpal tunnel syndrome: A review of the recent literature. Open Orthop J. 2012;6:69–76. 10.2174/1874325001206010069. PMC3314870 22470412

[3] Bagatur AE, Zorer G. The carpal tunnel syndrome is a bilateral disorder. J Bone Jt Surg Br. 2001;83:655–8. 10.1302/0301-620x.83b5.11350. 11476299

[4] Franklin GM, Friedman AS. Work-related carpal tunnel syndrome: Diagnosis and treatment guideline. Phys Med Rehabil Clin N Am. 2015;26:523–37. 10.1016/j.pmr.2015.04.003. 26231963

[5] Georgiew F. Testy prowokacyjne stosowane w diagnostyce zespołu cieśni nadgarstka; 2007. p. 11.

[6] Evaluation of Boston questionnaire applied at late pos-operative period of carpal tunnel syndrome operated with the paine retinaculatome through palmar port n.d. http://www.scielo.br/scielo.php?script=sci_arttext&pid=S1413-78522006000300002&lng=en&nrm=iso&tlng=en (accessed November 21, 2019).

[7] Hudak PL, Amadio PC, Bombardier C. Development of an upper extremity outcome measure: The DASH (disabilities of the arm, shoulder and hand) [corrected]. The upper extremity collaborative group (UECG). Am J Ind Med. 1996;29:602–8. 10.1002/(SICI)1097-0274(199606)29:6<602:AID-AJIM4>3.0.CO;2-L. 8773720

[8] Sulewski A. Wspó3czesne pogl1dy dotycz1ce leczenia neuropatii uciskowych w obrêbie nerwów koñczyny górnej n.d.:6. 19274866

[9] Georgiew F, Otfinowska E, Adamczyk T. Diagnostic tests used in diagnosis of the carpal tunnel syndrome. Rehabilitacja Medyczna. 2008;12:24–35.

[10] Kohara N. [Clinical and electrophysiological findings in carpal tunnel syndrome]. Brain Nerve. 2007;59:1229–38. 18044199

[11] Werner RA, Andary M. Carpal tunnel syndrome: Pathophysiology and clinical neurophysiology. Clin Neurophysiol. 2002;113:1373–81. 10.1016/s1388-2457(02)00169-4. 12169318

[12] Błachnio J, Bogdan M. Podstawy termowizji i jej zastosowanie w badaniach medycznych. Zesz Nauk Politechniki Białostockiej Budowa i Eksploatacja Masz. 2004;Z. 12:7–17.

[13] Bauer J, Dereń E. Standaryzacja badań termograficznych w medycynie i fizykoterapii. Acta Bio-Optica et Informatica Medica Inżynieria Biomedyczna. vol. 20; 2014.

[14] Nowakowski A, Kaczmarek M, Ruminski J, Hryciuk M, Renkielska A, Grudzinski J, et al. Medical applications of model based dynamic thermography. Proc SPIE. 2001;4360:492–503.

[15] Meyers S, Cros D, Sherry B, Vermeire P. Liquid crystal thermography: Quantitative studies of abnormalities in carpal tunnel syndrome. Neurology. 1989;39:1465–9. 10.1212/wnl.39.11.1465. 2812323

[16] Chung MS, Gong HS, Baek GH. Raynaud’s phenomenon in idiopathic carpal tunnel syndrome: Postoperative alteration in its prevalence. J Bone Jt Surg Br. 2000;82:818–9. 10.1302/0301-620x.82b6.10991. 10990303

[17] Keith MW, Masear V, Chung K, Maupin K, Andary M, Amadio PC, et al. Diagnosis of carpal tunnel syndrome. J Am Acad Orthop Surg. 2009;17:389–96. 10.5435/00124635-200906000-00007. PMC5175465 19474448

[18] Gniadek M, Trybus M. Carpal tunnel syndrome-etiology and treatment. Prz Lek. 2016;73:520–4. 29677425

[19] Aminoff MJ. Involvement of peripheral vasomotor fibres in carpal tunnel syndrome. J Neurol Neurosurg Psychiatry. 1979;42:649–55. 10.1136/jnnp.42.7.649. PMC490280 479905

[20] Verghese J, Galanopoulou AS, Herskovitz S. Autonomic dysfunction in idiopathic carpal tunnel syndrome. Muscle Nerve. 2000;23:1209–13. 10.1002/1097-4598(200008)23:8<1209:aid-mus8>3.0.co;2-#. 10918257

[21] Kiylioglu N, Akyol A, Guney E, Bicerol B, Ozkul A, Erturk A. Sympathetic skin response in idiopathic and diabetic carpal tunnel syndrome. Clin Neurol Neurosurg. 2005;108:1–7. 10.1016/j.clineuro.2004.12.003. 16311138

[22] Mondelli M, Vecchiarelli B, Reale F, Marsili T, Giannini F. Sympathetic skin response before and after surgical release of carpal tunnel syndrome. Muscle Nerve. 2001;24:130–3. 10.1002/1097-4598(200101)24:1<130:aid-mus20>3.0.co;2-h. 11150978

[23] Kuwabara S, Tamura N, Yamanaka Y, Misawa S, Isose S, Bae JS, et al. Sympathetic sweat responses and skin vasomotor reflexes in carpal tunnel syndrome. Clin Neurol Neurosurg. 2008;110:691–5. 10.1016/j.clineuro.2008.04.004. 18485585

[24] Wilder-Smith EPV, Fook-Chong S, Chew SE, Chow A, Guo Y. Vasomotor dysfunction in carpal tunnel syndrome. Muscle Nerve. 2003;28:582–6. 10.1002/mus.10475. 14571460

[25] Papež BJ, Palfy M. EMG vs thermography in severe carpal tunnel syndrome. EMG Methods for Evaluating Muscle and Nerve Function; 2012. 10.5772/26120.

[26] Jesensek Papez B, Palfy M, Turk Z. Infrared thermography based on artificial intelligence for carpal tunnel syndrome diagnosis. J Int Med Res. 2008;36:1363–70. 10.1177/147323000803600625. 19094447

[27] Jesensek Papez B, Palfy M, Mertik M, Turk Z. Infrared thermography based on artificial intelligence as a screening method for carpal tunnel syndrome diagnosis. J Int Med Res. 2009;37:779–90. 10.1177/147323000903700321. 19589261

[28] Zivcak J, Hudak R, Tkáčová M, Švehlík J. A role of thermography in the diagnostics of carpal tunnel syndrom. Acta Mechanica Slov. 2010;14. 10.2478/v10147-011-0017-9.

[29] Ming Z, Siivola J, Pietikainen S, Närhi M, Hänninen O. Postoperative relieve of abnormal vasoregulation in carpal tunnel syndrome. Clin Neurol Neurosurg. 2007;109:413–7. 10.1016/j.clineuro.2007.02.014. 17400369

[30] Ming Z, Zaproudina N, Siivola J, Nousiainen U, Pietikainen S. Sympathetic pathology evidenced by hand thermal anomalies in carpal tunnel syndrome. Pathophysiology. 2005;12:137–41. 10.1016/j.pathophys.2005.05.002. 16009539

[31] Hong YP, Ryu KS, Cho BM, Oh SM, Park SH. Evaluation of thermography in the diagnosis of carpal tunnel syndrome: Comparative study between patient and control groups. J Korean Neurosurg Soc. 2006;39:423.

